# Efficacy and Safety of Apatinib for the Treatment of Advanced or Recurrent Cervical Cancer: A Single-Arm Meta-Analysis Among Chinese Patients

**DOI:** 10.3389/fphar.2022.843905

**Published:** 2022-08-11

**Authors:** Da Huang, Qionghua He, Lingyun Zhai, Jiayu Shen, Fei Jing, Huanhuan Chen, Xiaoqing Zhu, Jianwei Zhou

**Affiliations:** ^1^ Department of Gynecology, The Second Affiliated Hospital, School of Medicine, Zhejiang University, Hangzhou, China; ^2^ Department of Obstetrics, The Second Affiliated Hospital, School of Medicine, Zhejiang University, Hangzhou, China

**Keywords:** apatinib, recurrent/metastatic cervical cancer, objective response rate, disease control rate, adverse events

## Abstract

**Background:** Although various effective compounds for the second- and third-line treatment of advanced or recurrent cervical cancer improved the overall survival, the optimal regimen remains controversial. Previous studies revealed that apatinib had extensive anti-tumor activities. However, almost all studies on apatinib in recurrent cervical cancer are non-randomized controlled trials with small sample sizes, different first-line treatments, and uncontrolled statistical analysis, which may result in a lack of effective metrics to evaluate the efficacy and safety of apatinib. Here, this meta-analysis aims to evaluate the efficacy and safety of apatinib in patients with advanced or recurrent cervical cancer.

**Methods:** PubMed, Embase, the Cochrane Library, and Web of Science databases were systematically searched for relevant studies. Outcomes including overall response rate (ORR), disease control rate (DCR), progression-free survival (PFS), overall survival (OS), and adverse events (AEs) were extracted for further analysis.

**Results:** Seven studies involving 243 patients were enrolled in this meta-analysis. In terms of tumor response, the pooled ORR and DCR were 22.9% and 68.6%, respectively. With regard to survival analysis, the pooled PFS and OS were 5.19 months and 10.63 months, respectively. The most common treatment-related adverse events of apatinib were hand–foot syndrome (all grade: 39.6%, ≥grade III: 7.5%), hypertension (all grade: 34.5%, ≥grade III: 9.2%), and fatigue (all grade: 28.0%, ≥grade III: 5.1%).

**Conclusions:** In summary, this meta-analysis demonstrated that apatinib has promising efficacy and safety for patients with advanced or recurrent cervical cancer.

**Systematic Review Registration:** https://inplasy.com/inplasy-2022-7-0049/, identifier INPLASY202270049

## Introduction

Cervical cancer is the fourth most common cancer in women worldwide, with approximately 570,000 new cases and 312,000 deaths annually (Bray et al., 2018). Despite early cervical cancer screening and subsequent surgical removal of the precancerous lesion help reducing the cancer incidence and mortality, more than 70% of cervical cancer cases in developing countries are diagnosed at an advanced stage of the disease (Sahasrabuddhe et al., 2012; Liu et al., 2019). Compared to local or early cervical cancer, outcomes for patients with advanced or recurrent disease are poor. Previous studies have shown that the 5-year survival rate for advanced cervical cancer is less than 40%, and for patients with stage IV, the survival rate remains only 5–15% (Tewari and Monk, 2005). Though platinum-based chemotherapies are still the cornerstone treatment for the majority of these patients, the prognosis is unsatisfactory due to multidrug resistance and side effects. Also, follow-up treatments are just palliative for the purpose of prolonging or maintaining patients’ survival (Elst et al., 2007; Pfaendler and Tewari, 2016). Hence, it is urgent to develop new methods to improve long-term disease control for advanced or recurrent cervical cancer.

Against this background, anti-angiogenic therapy is an attractive and promising treatment option for patients with advanced or recurrent cervical cancer. The results from the phase III GOG240 trial directly recommended that the combination of platinum-based chemotherapy and bevacizumab was the standard frontline treatment in advanced or recurrent cervical cancer, which showed an improvement in overall survival from 13.3 to 17 months (Tewari et al., 2014a). However, other angiogenesis inhibitors have also been tested, but there is no consensus on the benefit of second- and third-line chemotherapy in this disease. Apatinib is an oral, small-molecule tyrosine kinase inhibitor that targets vascular endothelial growth factor receptor 2(VEGFR-2). Several clinical trials have shown that apatinib could improve tumor response and survival in various cancer types of middle-late solid tumors (Hu et al., 2014; Lan et al., 2018; Qin et al., 2021; Zhao et al., 2021). However, almost all studies on apatinib in recurrent cervical cancer are non-randomized controlled trials with small sample sizes, different first-line treatments, and uncontrolled statistical analysis, which may result in a lack of effective measurement for evaluating the efficacy and safety of apatinib. Thus, this meta-analysis was carried out to evaluate the efficacy and safety of apatinib in advanced or recurrent cervical cancer patients and hoped that the results of this study might provide more options for clinical treatments.

## Materials and Methods

### Search Strategy

Four databases (PubMed, Embase, the Cochrane Library, and Web of Science) were comprehensively searched for relevant studies. The date of the last search was 25 June 2021. The following MeSH and free words were used in the searches: “Uterine Cervical Neoplasms OR Uterine Cervical Cancer OR Cervical Cancer OR Cancer of the Cervix” AND “Apatinib OR rivoceranib mesylate OR YN968D1 OR rivoceranib OR apatinib mesylate”. The language was confined to English. In addition, we evaluated the references of included articles and selected more relevant studies.

### Selection Criteria

Studies were included in this meta-analysis if they met the following inclusion criteria: 1) population: patients were diagnosed with advanced or recurrent cervical cancer, irrespective of the subtype; 2) intervention: patients were treated with apatinib, either with single-agent therapy or in combination with chemotherapy; 3) study type: phase II clinical trials or retrospective analysis; and 4) outcomes: patients were reported with interested clinical tumor outcomes, including objective response rate (ORR), disease control rate (DCR), progression-free survival (PFS), overall survival (OS), and adverse events (AEs). The tumor response was evaluated using the Response Evaluation Criteria in Solid Tumors (RECIST) version1.1 (Eisenhauer et al., 2009). Toxic effects were evaluated for their incidence and severity using the Common Terminology Criteria for Adverse Events (CTCAE) (Trotti et al., 2003). The exclusion criteria were as follows: 1) animal experiments, cell research, reviews, meta-analyses, duplicates, case reports, or letters were not taken into consideration; and 2) studies with patient number less than 10 were excluded. Two investigators independently identified potential eligible articles through inclusion and exclusion criteria. Any disagreement regarding study inclusion was resolved between these two or with a third investigator.

### Data Extraction and Quality Assessment

The required data from all included studies were independently extracted by two investigators, and the quality assessment of the studies was performed afterward. The extracted characteristics were summarized as follows: authors, publication year, nation, sample size, prior therapeutic regimen, median age, median follow-up, and reported endpoints. Indexes for clinical and safety outcomes included ORR, DCR, OS, PFS, the incidence of any AEs, and ≥grade 3 AEs. Also, two investigators independently assessed and extracted the required data from all included studies. The Newcastle–Ottawa Scale (NOS) was used to evaluate the quality of including non-controlled trials (Stang, 2010). Also, the retrospective studies were assessed by the JBI Critical Appraisal Checklist for Case Series (TJB, 2016).

### Statistical Analysis

All data in this meta-analysis were analyzed with STATA 14.2 software (StataCorp LP, College Station, TX, United States). Heterogeneity was measured using the chi-squared test and I^2^ statistic. *p* < 0.1 indicated a statistically significant difference. If significant heterogeneity (*p*-value <0.1 and I^2^>50%) existed, a random-effect model was performed. Otherwise, the fixed-effects model was used (Yao et al., 2021). Moreover, sensitivity analysis was performed to analyze the stability and reliability of the pooled results. Finally, a potential publication bias was accessed by Begg’s and Egger’s tests.

## Result

### Study Selection

**FIGURE 1:**
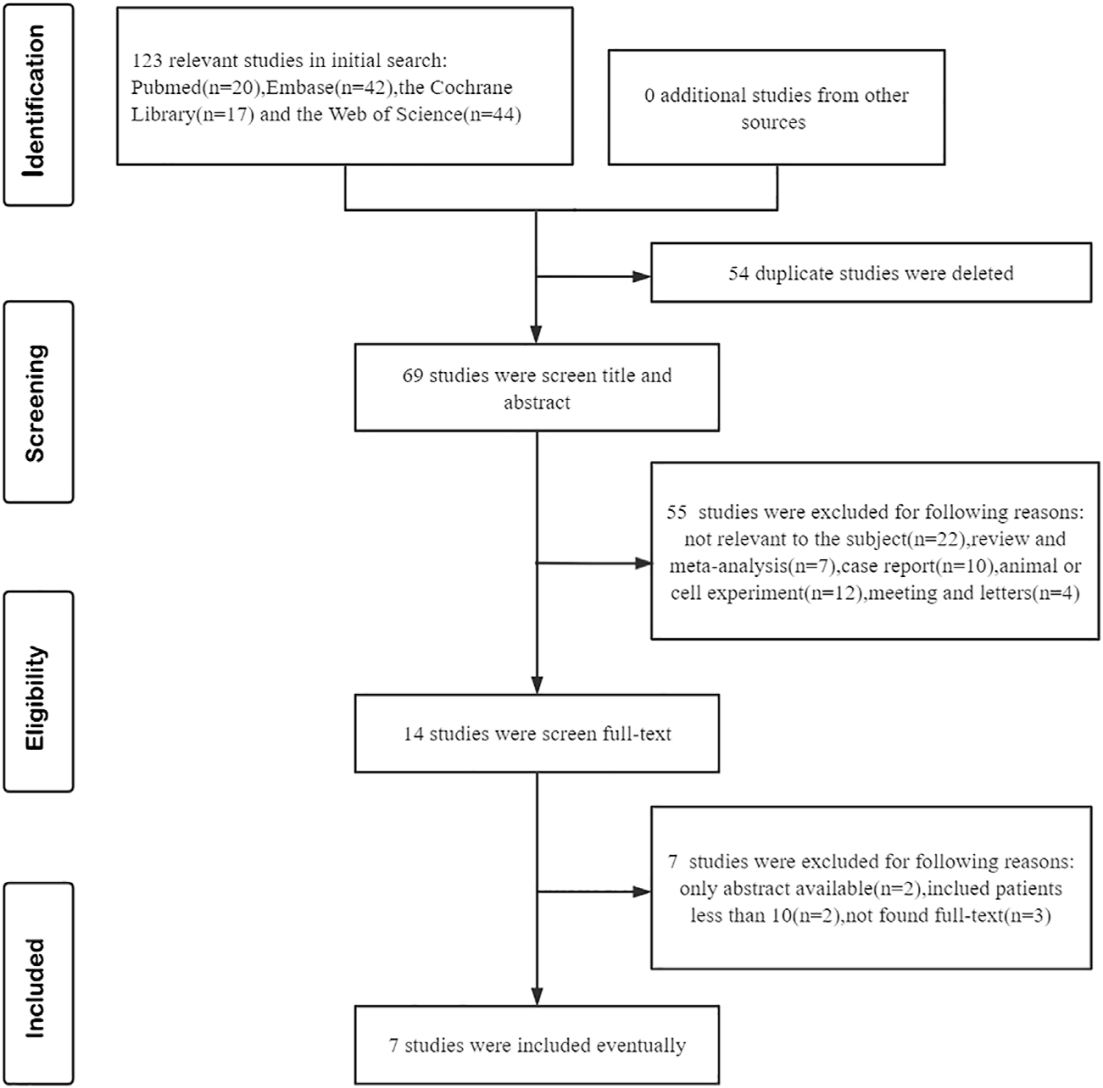
Flow diagram of meta-analysis for inclusion/exclusion of studies.

The initial search yielded a total of 123 published relevant studies from four databases (PubMed = 20, Embase = 42, Web of Science = 44, and Cochrane Library = 17). About 14 studies were retained after removing duplicates and screening the titles and abstracts. Then, the remaining full-text articles were assessed carefully, and seven studies were excluded due to unavailable full text, small sample size, or non-chemotherapy drugs. Finally, seven studies with a total of 243 patients met the inclusion criteria and were included in this meta-analysis (Su et al., 2019; Yu et al., 2019; Li et al., 2020; Xia et al., 2020; Xiao et al., 2020; Zhang et al., 2020; Yang et al., 2021).The flowchart of the selection process is shown in Figure 1. The details of each included study are described in Table 1.

**TABLE 1:** Characteristics of the studies included in the meta-analysis.

Study, year	Nation	Sample size	Mean age, years	Median follow-up, months	Intervention	Prior therapy	Histology	Endpoints
Xie et al., 2019	China	25	55 (26–73)	NR	Apatinib	CT	Cervical squamous cell carcinoma	ORR, DCR, PFS, OS, and AEs
Zhu et al., 2019	China	26	58 (36–72)	5.0 (3–24)	Apatinib	CT + RT; CT; RT	Squamous cell carcinoma, adenocarcinoma, and adenosquamous carcinoma	ORR, DCR, PFS, OS, and AEs
Cheng et al., 2020	China	48	58 (32–75)	14.5 (5.5–20.5)	Apatinib	CT + RT; Surgery; CT; RT	Squamous carcinoma, adenocarcinoma, and adenosquamous carcinoma	ORR, DCR, PFS, OS, and AEs
Yu et al., 2020	China	20	47.2 (36–60)	14.0 (5.9–21.3)	Apatinib	CT; CT + bevacizumab	Squamous cell carcinoma and adenocarcinoma	ORR, DCR, PFS, OS, and AEs
Zhao et al., 2020	China	42	52 (28–70)	13	Apatinib	CT + RT; CT + bevacizumab	Squamous cell carcinoma, adenosquamous cell carcinoma, and adenocarcinoma	ORR, DCR, PFS, OS, and AEs
Yang et al., 2021	China	53	<50 (n = 20)>50 (n = 33)	NR	Apatinib	RT; surgery	Squamous cell carcinoma and adenosquamous cell carcinoma	ORR, DCR, PFS, OS, and AEs
Wu et al., 2020	China	29	48 (30–63)	18	Apatinib	CT + RT; CT; RT	Cervical squamous cell carcinoma, cervical adenocarcinoma, and cervical small cell cancer	ORR, DCR, PFS, OS, and AEs

NR, not reported; CT, chemotherapy; RT, radiotherapy; ORR, overall response rate; DCR, disease control rate; OS, overall survival; PFS, progression-free survival; AEs, adverse events.

### Quality Assessment

Two non-randomized studies were assessed using the Newcastle–Ottawa Scale (NOS), which categorized studies into three dimensions based on eight items, including population election, comparability, and outcome (cohort studies) or exposure (case–control studies) evaluation (Stang, 2010). Five retrospective studies were assessed using the JBI Critical Appraisal Checklist for Case Series, which contains ten items that assess the quality of case reports from the selection of cases, disease or health problem evaluation, and presentation of case data. The quality assessment details are shown in Table 2.

**TABLE 2:** Quality assessment of the studies included in the meta-analysis.

Study	Q1	Q2	Q3	Q4	Q5	Q6	Q7	Q8	Q9	Q10	Total
JBI Critical Appraisal Checklist for Case Series for included retrospective studies											
Xie et al., 2019	2	0	2	2	2	0	2	2	2	2	16
Zhu et al., 2019	2	0	2	2	2	0	2	2	2	2	16
Cheng et al., 2020	2	0	2	2	2	0	2	2	2	2	16
Yang et al., 2021	2	0	2	2	2	0	2	2	2	2	16
Wu et al., 2020	2	0	2	2	2	0	2	2	2	2	16
Study	Ⅰ	Ⅱ	Ⅲ	Ⅳ	Ⅴ	Ⅵ	Ⅶ	Ⅷ			Total
Newcastle–Ottawa Scale (NOS) for included non-randomized studies											
Yu et al., 2020	1	0	1	1	0	1	0	1			5
Zhao et al., 2020	1	0	1	1	0	1	0	1			5

Numbers Q1-Q10 in heading signified: Q1, were there clear criteria for inclusion in the case series? Q2, was the condition measured in a standard, reliable way for all participants included in the case series? Q3, were valid methods used for identification of the condition for all participants included in the case series? Q4, did the case series have consecutive inclusion of participants? Q5, did the case series have complete inclusion of participants? Q6, was there clear reporting of the demographics of the participants in the study? Q7, was there clear reporting of clinical information of the participants? Q8, were the outcomes or follow-up results of cases clearly reported? Q9, was there clear reporting of the presenting site(s)/clinic(s) demographic information? Q10, was statistical analysis appropriate?

Numbers I-Ⅷ in heading signified: Ⅰ, representatives of the exposed cohort; Ⅱ, selection of the non-exposed cohort; Ⅲ, ascertainment of exposure; Ⅳ, demonstration that outcome of interest was present at the start of the study; Ⅴ, comparability of cohorts on the basis of the design or analysis; Ⅵ, assessment of the outcome; Ⅶ, was follow-up long enough for outcomes to occur? Ⅷ, adequacy of follow-up of cohorts.

### Tumor Response

**FIGURE 2:**
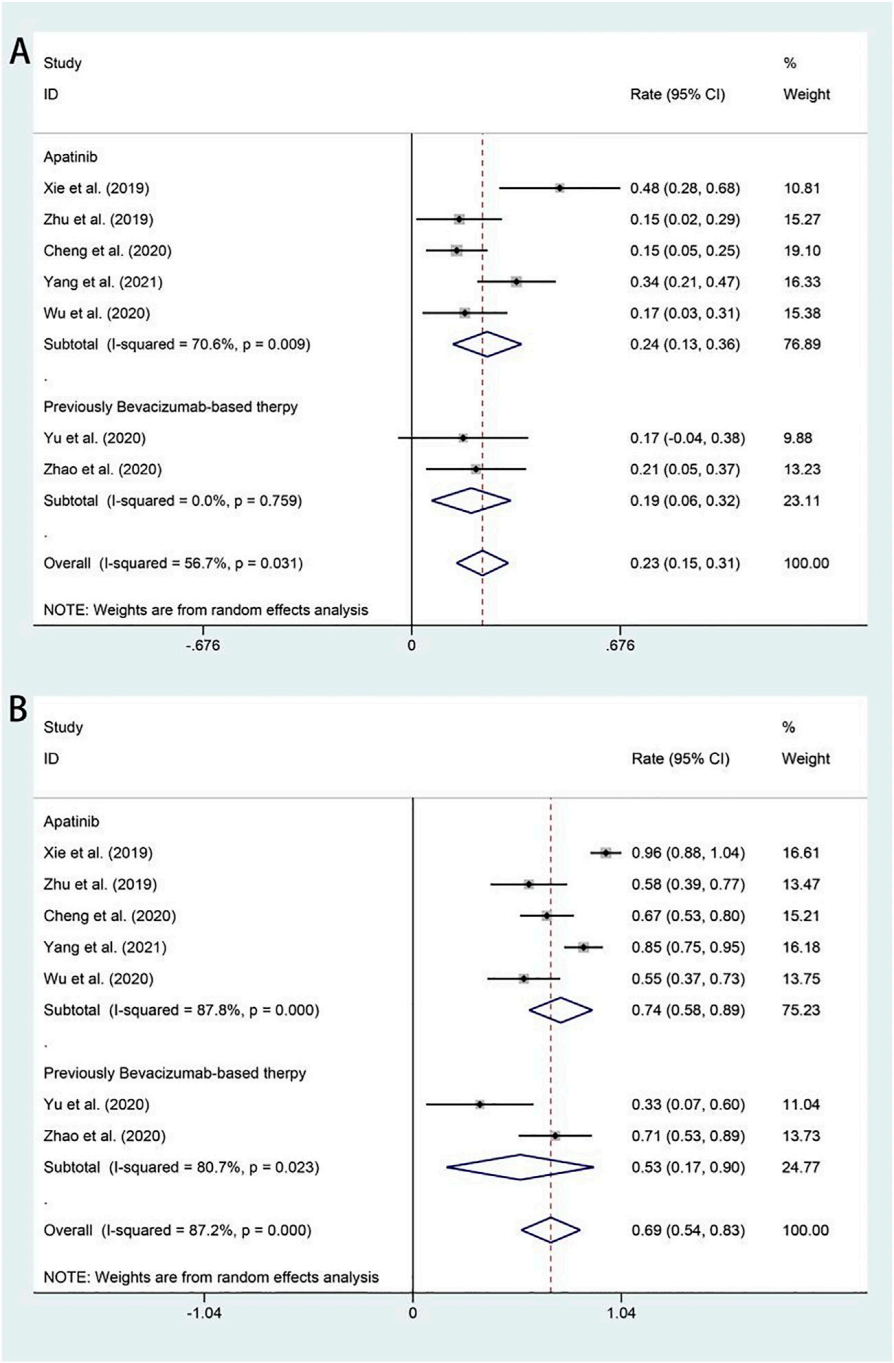
Forest plot about the pooled results of ORR **(A)** and DCR **(B)** in total by the treatment regimen subgroup. ORR, overall response rate; DCR, disease control rate.

All studies included in the analysis reported the efficacy response of apatinib for advanced or recurrent cervical cancer. The ORRs across the studies varied from 19 to 24%. The random-effects model was used because of significant heterogeneity (I^2^ = 56.7%, *p* = 0.031). The analysis showed a pooled ORR of 22.9% (95% CI: 14.5%–31.3%), and the ORR was further analyzed according to different apatinib treatment regimens. Subgroup analysis revealed that the pooled ORR in patients who received bevacizumab as first-line chemotherapy was 19.3% (95% CI: 6.4%–32.2%). Otherwise, the ORR of patients in apatinib monotherapy was 24.5% (95% CI: 13.3%–35.6%) (Figure 2A). All studies also included available data on DCR, and the pooled DCR was 68.6% (95% CI: 53.9%–83.3%), with significant heterogeneity (I^2^ = −87.2%, *p* = 0.000). Subgroup analysis showed that the pooled DCR in patients who received bevacizumab as first-line chemotherapy was 53.4% (95% CI: 16.7%–90.1%). Otherwise, the ORR of patients in apatinib monotherapy achieved a higher pooled DCR of 73.6% (95% CI: 57.8%–89.3%) (Figure 2B).

### Survival

**FIGURE 3:**
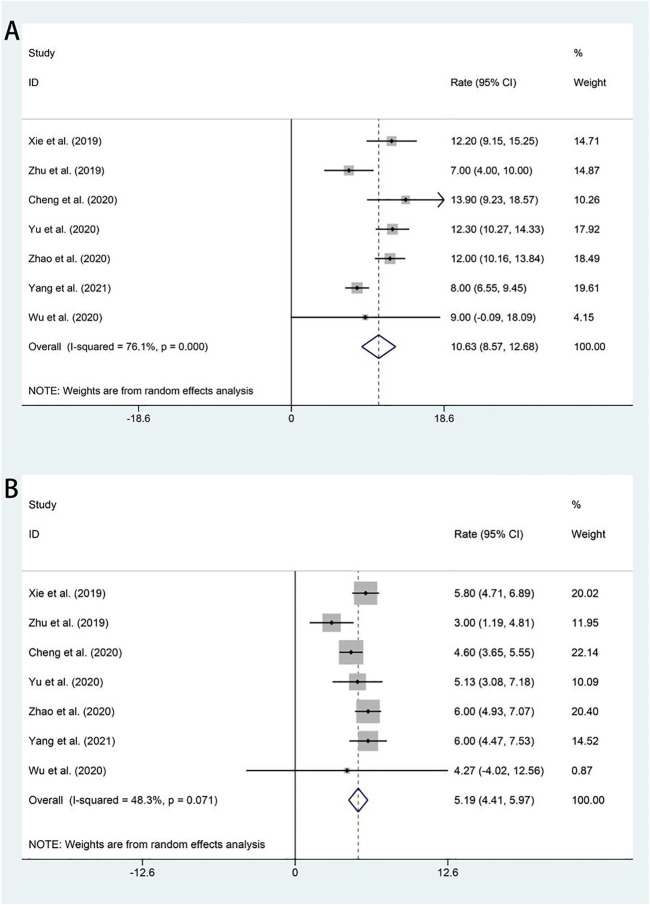
Forest plot about the pooled results of OS **(A)** and PFS **(B)** in total by the treatment regimen. OS, overall survival; PFS, progression-free survival.

All studies included in the analysis reported OS and PFS for all patients after administration of apatinib. In the random-effects model (I^2^ = 76.1%, *p* = 0.000), the pooled median OS was 10.63 months (95% CI 8.57–12.68 months), as shown in Figure 3A. With regard to PFS, the fixed-effects model was performed (I^2^ = 48.3%, *p* = 0.071), and the results showed that the pooled median PFS was 5.25 months (95% CI: 4.73–5.76 months) (Figure 3B).

### Toxicities

The most common AEs (all grades and grade ≥ III) associated with apatinib in treating advanced or recurrent cervical cancer were analyzed (Table 3 and Supplementary Figures S1, S2). Most patients went through grades 1–2 AEs and were well tolerated. The results also indicated the three most commonly reported adverse events, including hypertension, hand–foot syndrome, and fatigue, with an incidence of 34.5% (95% CI: 22.5%–46.4%), 39.6% (95% CI: 25.5%–53.7%), and 28.0% (95% CI: 15.9%–40.0%), respectively. Then, the most common hematologic toxicities included hemorrhage (15.7%, 95% CI: 1.5%–33.0%), neutropenia (15.5%, 95% CI: 2.8%–28.1%), and thrombocytopenia (8.2%, 95% CI: 2.6%–18.9%). Additionally, the incidence rates of gastrointestinal AEs, such as diarrhea and nausea, reached 22.4% (95% CI: 10.8%–34.0%). The incidence of grade ≥ III adverse events was significantly lower, rarely exceeding 10%. Even the most commonly reported incidences of AEs such as hypertension, hand–foot syndrome, and fatigue were only 9.2%, 7.5%, and 3.0%, respectively.

**TABLE 3:** Adverse events of the studies included in the meta-analysis.

AE	All grade	≥Grade III		
	ES, % (95 CI)	I^2^,%	ES, % (95 CI)	I^2^,%
Hand–foot syndrome	39.6 (25.5–53.7)	82.3	7.5 (2.7–12.3)	0
Hypertension	34.5 (22.5–46.4)	76.4	9.2 (3.6–14.7)	0
Proteinuria	15.0 (5.7–24.3)	77.6	3.0 (0.0–6.6)	0
Fatigue	28.0 (15.9–40.0)	76.9	5.1 (0–11.0)	44.8
Hemorrhage	15.7 (−1.5 to 33.0)	89.1	5.0 (0–11.8)	NA
Thrombocytopenia	8.2 (−2.6 to 18.9)	49.5	NA	NA
Diarrhea and nausea	22.4 (10.8–34.0)	73.5	9.3 (2.6–15.9)	20.4
Neutropenia	15.5 (2.8–28.1)	89.5	9.1 (2.9–15.3)	0

### Sensitivity Analysis

Sensitivity analysis was conducted by omitting one study at a time to assess its effect on pooled results. As indicated by the results of the analysis, all of the pooled results with 95% CIs were not remarkably influenced by any individual study. This demonstrated that the results of this meta-analysis were relatively reliable in total. The results of sensitivity analysis are shown in Supplementary Figure S3.

### Publication Bias

To ensure the validity of the meta-analysis results, Egger’s and Begg’s tests were used to estimate the publication bias. The test results were consistent with most of the results except the ORR (Egger’s test: 0.001; Begg’s test: 0.035). With regard to safety, we considered that the publication bias exists for proteinuria, fatigue and diarrhea, and nausea (Supplementary Table S1).

## Discussion

Tumor angiogenesis is heterogeneous and has different histopathological characteristics of normal blood vessels, and it creates a tumor microenvironment characterized by hypoxia, hyperpermeability, poor perfusion, and acidosis in tissues, which promotes tumor growth, invasiveness, and metastasis (Matsumoto et al., 2011; Viallard and Larrivée, 2017). Anti-angiogenic therapy could remodel the structure and function of abnormal vessels, which transiently normalize angiogenesis by decreasing tumor vessel hyperpermeability and increasing tumor perfusion and blood supply, thus improving the hypoxia and acidosis environments and enhancing the benefits of chemotherapeutic drugs and radiotherapy (Lin and Sessa, 2004; Goel et al., 2011; Li et al., 2021). So far, the vital role of the vascular endothelial growth factor (VEGF)/VEGF-receptor (VEGFR) signaling pathway in the development and progression of advanced cervical cancer has been identified (Dobbs et al., 1997; Tomao et al., 2014; Garcia et al., 2020). Bevacizumab, an anti-VEGF monoclonal antibody, is one of the most extensively researched anti-angiogenetic treatments. A phase II clinical trial assessed the efficacy of bevacizumab monotherapy in patients with recurrent or metastatic cervical cancer, and the results showed that the median PFS and OS were 3.40 and 7.29 months, respectively, and the ORR was 11%. But the pathological type involved in this study was only squamous cell carcinoma (SCC); hence the clinical benefits of cervical adenocarcinoma remained to be investigated (Monk et al., 2009). Though, to some extent, anti-angiogenic therapy alone can bring survival benefits to patients, the effect was modest. Therefore, the combination of anti-angiogenic and cytotoxic compounds was expected to achieve better efficacy results. There is a large amount of published literature proving that bevacizumab has survival benefits when combined with platinum-based chemotherapy for advanced and recurrent cervical cancer. Also, the combination therapy showed no significant deterioration in health-related quality of life. Despite the use of bevacizumab in cervical cancer showed efficacy, its high cost also limits its use (Tewari et al., 2014b; Fisher and Schefter, 2015; Rosen et al., 2017). Previous studies also evaluated other alternative anti-angiogenic strategies, including pazopanib, lapatinib, gefitinib, and sunitinib (Goncalves et al., 2008; Mackay et al., 2010; Monk et al., 2010), but the results were hardly satisfactory. The ORRs of pazopanib, lapatinib, gefitinib, and sunitinib were 9.5%, 5.1%, 0%, and 0%, respectively. Also, DCRs of pazopanib, lapatinib, gefitinib and sunitinib were 52.7%, 48.7%, 20%, and 84.2%, respectively. From the perspective of survival data, the median PFS of pazopanib, lapatinib, and sunitinib was 18.1, 17.1 weeks, and 3.5 months, respectively. The median OS of pazopanib and lapatinib was 50.7 and 39.1 weeks, respectively.

Apatinib, another novel anti-angiogenic drug, showed satisfactory short-term effects in tumor treatment. It selectively binds to and inhibits VEGFR-2, leading to decreased vascular endothelial cell proliferation and tumor vascular assembly (Tian et al., 2011; Scott et al., 2015).Based on the results, apatinib was approved for the third-line treatment of advanced gastric patients in China (Li et al., 2013). In this meta-analysis, seven clinical studies with 243 patients were included to evaluate the efficacy and safety of apatinib in treating advanced or recurrent cervical cancer. The pooled analyses presented that apatinib exhibited efficacy and manageable safety with promising ORR, DCR, OS, and PFS. Despite the disease status, treatment of the cancer, and subtypes, the pooled results showed that the ORR and DCR were 22.9% and 68.6%, respectively, and the median OS and PFS were 10.63 months and 5.25 months, respectively. The subgroup analysis indicated that it was likely that initial use of anti-angiogenic inhibitors resulted in an increased ORR (24.5% vs. 19.3%) and DCR (73.6% vs. 53.4%). The aforementioned results demonstrated that apatinib monotherapy has a better effect in patients with advanced or recurrent cervical cancer. Considering that bevacizumab may affect the evaluation of apatinib efficacy, we reanalyzed two studies that used bevacizumab as first-line treatment. The results showed that the pooled ORR and DCR were 19.3% and 53.4%, respectively. However, because bevacizumab is not approved by the China Food and Drug Administration for the treatment of recurrent cervical cancer, the proportion of those patients in our study was relatively small, and this might exaggerate the response to apatinib. Nevertheless, multivariate Cox regression showed that first-line bevacizumab therapy was not associated with survival, which suggested that cross-resistance might not exist between apatinib and bevacizumab. Thus, patients might still respond to other VEGFR inhibitors after failure of first-line VEGF therapy (Xia et al., 2020). Furthermore, the role of bevacizumab or apatinib in advanced cervical cancer has been demonstrated, and we cannot help thinking that whether these anti-angiogenic inhibitors have a greater therapeutic effect in combination with radiotherapy. Interestingly, multiple preclinical studies observed an enhanced effect of the combinatorial approach, such as in locally advanced pancreatic cancer and rectal cancer (Mauceri et al., 1998; Crane et al., 2006; Czito et al., 2007). In cervical cancer, a prospective phase II trial was conducted to investigate the efficacy of bevacizumab with standard chemoradiotherapy for untreated locally advanced cervical carcinoma. Initial clinical results are encouraging, as association of bevacizumab with chemoradiotherapy resulted in a 3-year OS of 81.3%, disease-free survival (DFS) of 68.7%, and locoregional failure (LRF) of 23.2% (Schefter et al., 2014). Also, multivariate analysis demonstrated that the biomarker of VEGF was an independent predictor of poor prognosis in advanced cervical cancer patients after treating with radiotherapy (Gaffney et al., 2003). Since there is evidence that adequate tumor vascularization increases tumor perfusion and oxygenation, thereby enhancing radiotherapy efficacy, more clinical trials are needed to evaluate the impact of anti-angiogenic inhibitors in combination with radiotherapy on the efficacy of cervical cancer. Except for the promising efficacy of apatinib in cervical cancer treatment, the safety of this anti-angiogenic drug is also encouraging. Our study showed that the most commonly reported AEs with the highest incidence were hypertension (34.5%), hand–foot syndrome (39.6%), and fatigue (28%). Most AEs were of grades 1–2, and the incidence of grade ≥ III adverse events was significantly lower, rarely exceeding 10%. Even the most commonly reported incidences of AEs such as hypertension, hand–foot syndrome, and fatigue were only 9.2%, 7.5%, and 3.0%, respectively. According to these results, the safety of apatinib in treating advanced or recurrent cervical cancer patients is acceptable.

Tumor immunotherapy has emerged as one of the hottest research fields in recent years. Currently, hundreds of clinical trials are being carried out to evaluate the efficacy of immune checkpoint inhibitors (ICIs) for tumor patients, and most of them result in prolonged OS, PFS, or higher ORR (Darvin et al., 2018; Motzer et al., 2019). A multicenter, single-arm phase II clinical trial reported that combining camrelizumab with apatinib yielded higher ORR and DCR (55.6% and 82.2%, respectively), and most AEs were manageable (Lan et al., 2020). This demonstrated that the effect of immunotherapy combined with anti-angiogenesis is encouraging and attractive in the treatment of cervical cancer. However, since the follow-up time was short and survival data was incomplete, the benefits of immunotherapy combined with anti-angiogenic therapy on extended survival still need to be explored. Based on the currently published clinical data, the programmed death-1 (PD-1) pembrolizumab conferred a satisfactory outcome, with three complete and nine partial responses of ORR being 12.2% and thirty patients stable disease of DCR being 30.6% (Chung et al., 2019). The phase II clinical study responses were only seen in patients with programmed death-1(PD-L1) + tumors. Interestingly, PD-L1 expression is observed in 34.4–96% of cervical cancer tissues and programmed death-ligand 1 (PD-L1) expression varies from 51% to 88%, particularly in squamous cell carcinoma (SCC) (Mezache et al., 2015; Enwere et al., 2017; Reddy et al., 2017). The results led to FDA approval of pembrolizumab as a second-line treatment in this patient population. Nivolumab, another PD-L1 ICI, is approved for the treatment of various cancers. The effects of nivolumab were studied in 19 patients in the phase 1–2 study CheckMate358, and the ORR was 26.3%, regardless of PD-L1 expression, with a DCR of 68% (Naumann et al., 2019). In the meantime, several clinical trials have already evaluated the efficacy of ICIs combined with anti-angiogenic therapy in the recurrent or metastatic setting. It was reported that atezolizumab plus bevacizumab achieved no confirmed response in advanced cervical cancer (Friedman et al., 2020), but camrelizumab plus apatinib therapy achieved an ORR of 55.6% for all histological subtypes of cervical cancer (Lan et al., 2020). Despite the contradictions in the data, and camrelizumab or atezolizumab and apatinib, which represent two completely different therapy mechanisms, each of them has been proved to have promising anti-tumor activity. However, to prove whether the combination of immune checkpoint inhibitors and anti-angiogenic inhibitors or monotherapy can prolong the overall prognosis survival time of those patients needs more clinical trials. Fortunately, more and more PD-1/PD-L1 inhibitors are being evaluated in other cervical cancer trials (Cohen et al., 2020).

An phase III randomized trial is comparing PFS and OS among patients receiving standard platinum-based chemotherapy plus pembrolizumab compared to standard therapy plus placebo (Colombo et al., 2021). Another phase III randomized trial is exploring OS among patients treated with cisplatin, paclitaxel, and bevacizumab with and without atezolizumab (Grau et al., 2020). Avelumab and durvalumab are also being explored in clinical trials. There is a reason to believe that it will bring us surprising results in the near future (Thigpen et al., 1981; Thigpen et al., 1983; Xiong et al., 2019).

There were some limitations in the current meta-analysis. First, there was high heterogeneity that existed among the included studies. We only performed a subgroup analysis of the prior treatment regimens. Other factors, such as baseline characteristics and histological classification, could also result in heterogeneity. Second, the included studies were all non-controlled trials with small sample size, and thus we only evaluated the efficacy and risk without definite conclusions. Third, due to the lack of sufficient pathological data of cervical cancer, we were unable to analyze the efficacy of apatinib for different histological types of cervical cancer. Further analysis of the efficacy of apatinib on different histological types of cervical cancer is still needed in the future to achieve accurate treatment. Fourth, the trials in this analysis included only Chinese patients and the number of patients was relatively small. Whether these results are consistent in other populations still needs further validation. Therefore, more large-scale RCTs should be designed to confirm the clinical role of apatinib in comparison to that of other drugs and the population.

## Conclusion

In summary, our meta-analysis demonstrates the efficacy and safety of apatinib in patients with advanced or recurrent cervical cancer, providing evidence for its future clinical application. However, since there are limited clinical data, future large-scale and multiple-center RCTs are required to confirm this conclusion.

## Data Availability

The original contributions presented in the study are included in the article/Supplementary Material; further inquiries can be directed to the corresponding author.

## Supplementary Material

Supplementary Figure S1 Forest plot about the pooled results of any-grade adverse events. **(A)** Hand–foot syndrome, **(B)** hypertension, **(C)** proteinuria, **(D)** fatigue, **(E)** hemorrhage, **(F)** thrombocytopenia, **(G)** diarrhea and nausea, and **(H)** neutropenia.

Supplementary Figure S2 Forest plot about the pooled results of Grade III and higher adverse events. **(A)** Hand–foot syndrome, **(B)** hypertension, **(C)** proteinuria, **(D)** fatigue, **(E)** hemorrhage, **(F)** diarrhea and nausea, **(G)** neutropenia.

Supplementary Figure S3 Sensitivity analysis. **(A)** Sensitivity analysis for ORR; **(B)** sensitivity analysis for DCR; **(C)** sensitivity analysis for OS; **(D)** sensitivity analysis for PFS; **(E)** sensitivity analysis for hand–foot syndrome; **(F)** sensitivity analysis for hypertension; **(G)** sensitivity analysis for proteinuria; **(H)** sensitivity analysis for fatigue; **(I)** sensitivity analysis for hemorrhage; **(J)** sensitivity analysis for thrombocytopenia; **(K)** sensitivity analysis for diarrhea and nausea; and **(L)** sensitivity analysis for neutropenia. ORR, objective response rate; DCR, disease control rate; OS, overall survival; PFS, progression-free survival.

Supplementary Table S1 Begg’s and Egger’s tests of the results in the meta-analysis.
