# Supplementary material for: Efficacy and Safety of Apatinib for the Treatment of Advanced or Recurrent Cervical Cancer: A Single-Arm Meta-Analysis Among Chinese Patients
Source: Front Pharmacol. 2022 Aug 11;13:843905. doi: 10.3389/fphar.2022.843905 (PMC9403417; doi:10.3389/fphar.2022.843905)
Supplement: Supplementary file 1 [file DataSheet3.docx]

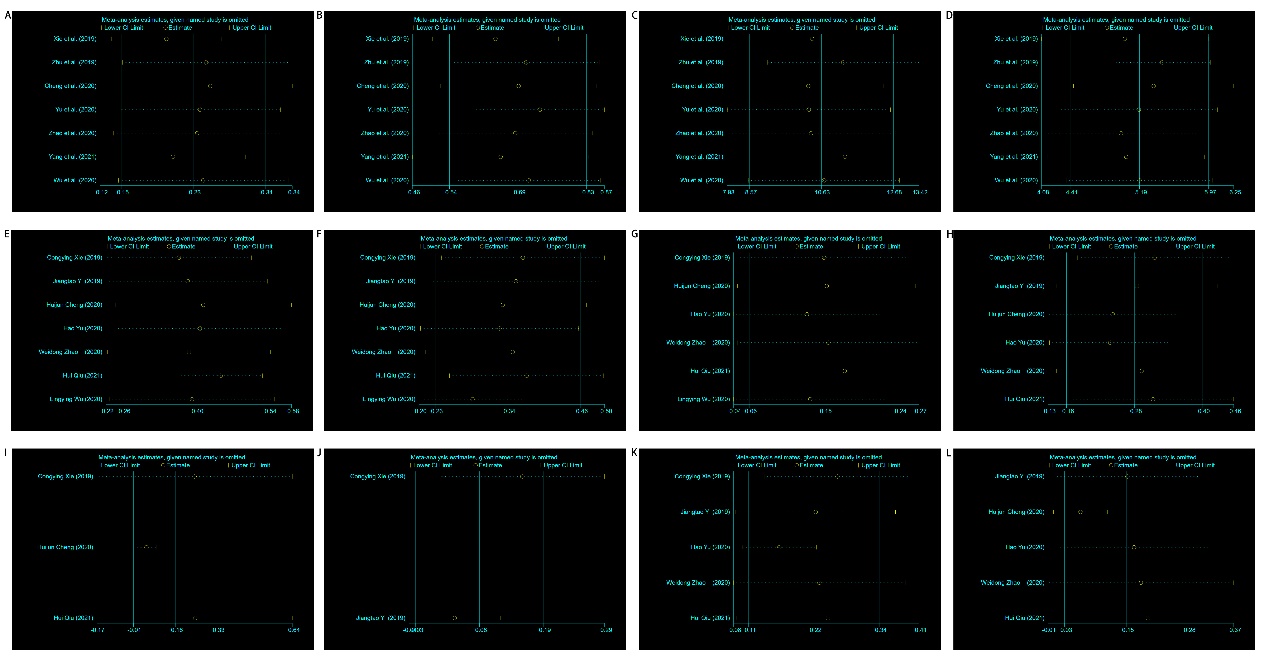


Figure. S3 Sensitivity analysis. (A) Sensitivity analysis for ORR; (B) Sensitivity analysis for DCR; (C) Sensitivity analysis for OS; (D) Sensitivity analysis for PFS; (E) Sensitivity analysis for hand-foot syndrome; (F) Sensitivity analysis for hypertension; (G) Sensitivity analysis for proteinuria; (H) Sensitivity analysis for fatigue; (I) Sensitivity analysis for hemorrhage; (J) Sensitivity analysis for thrombocytopenia; (K) Sensitivity analysis for diarrhea and nausea; (L) Sensitivity analysis for neutropenia. ORR, objective response rate; DCR, disease control rate; OS, overall survival; PFS, progression-free survival.
