# Supplementary material for: Efficacy and Safety of Apatinib for the Treatment of Advanced or Recurrent Cervical Cancer: A Single-Arm Meta-Analysis Among Chinese Patients
Source: Front Pharmacol. 2022 Aug 11;13:843905. doi: 10.3389/fphar.2022.843905 (PMC9403417; doi:10.3389/fphar.2022.843905)
Supplement: Supplementary file 2 [file DataSheet2.docx]

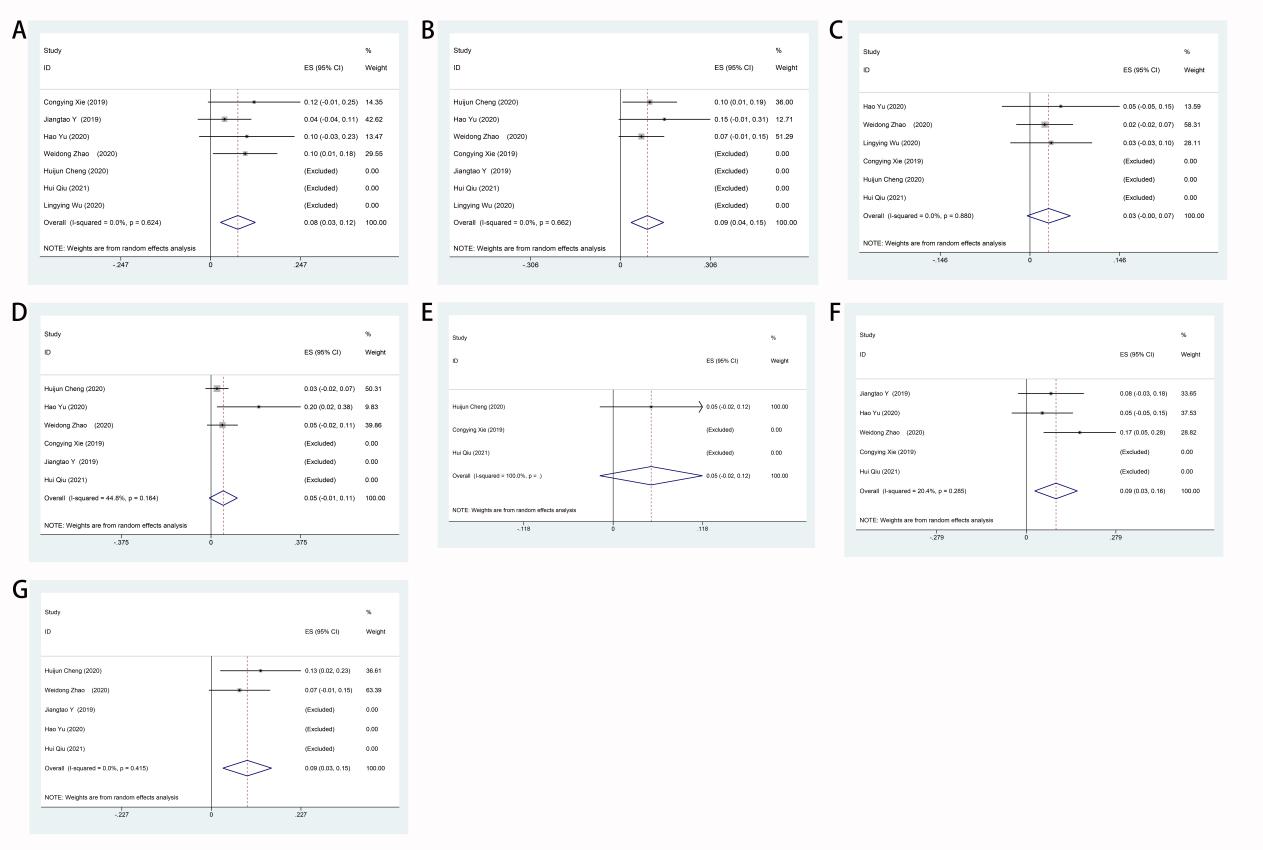


Figure. S2 The forest plot about the pooled results of Grade III and higher adverse events. (A) hand-foot syndrome (B) hypertension (C) proteinuria (D) fatigue (E) hemorrhage (F) diarrhea and nausea (G) neutropenia.
