# Supplementary material for: Efficacy and Safety of Apatinib for the Treatment of Advanced or Recurrent Cervical Cancer: A Single-Arm Meta-Analysis Among Chinese Patients
Source: Front Pharmacol. 2022 Aug 11;13:843905. doi: 10.3389/fphar.2022.843905 (PMC9403417; doi:10.3389/fphar.2022.843905)
Supplement: Supplementary file 3 [file DataSheet1.docx]

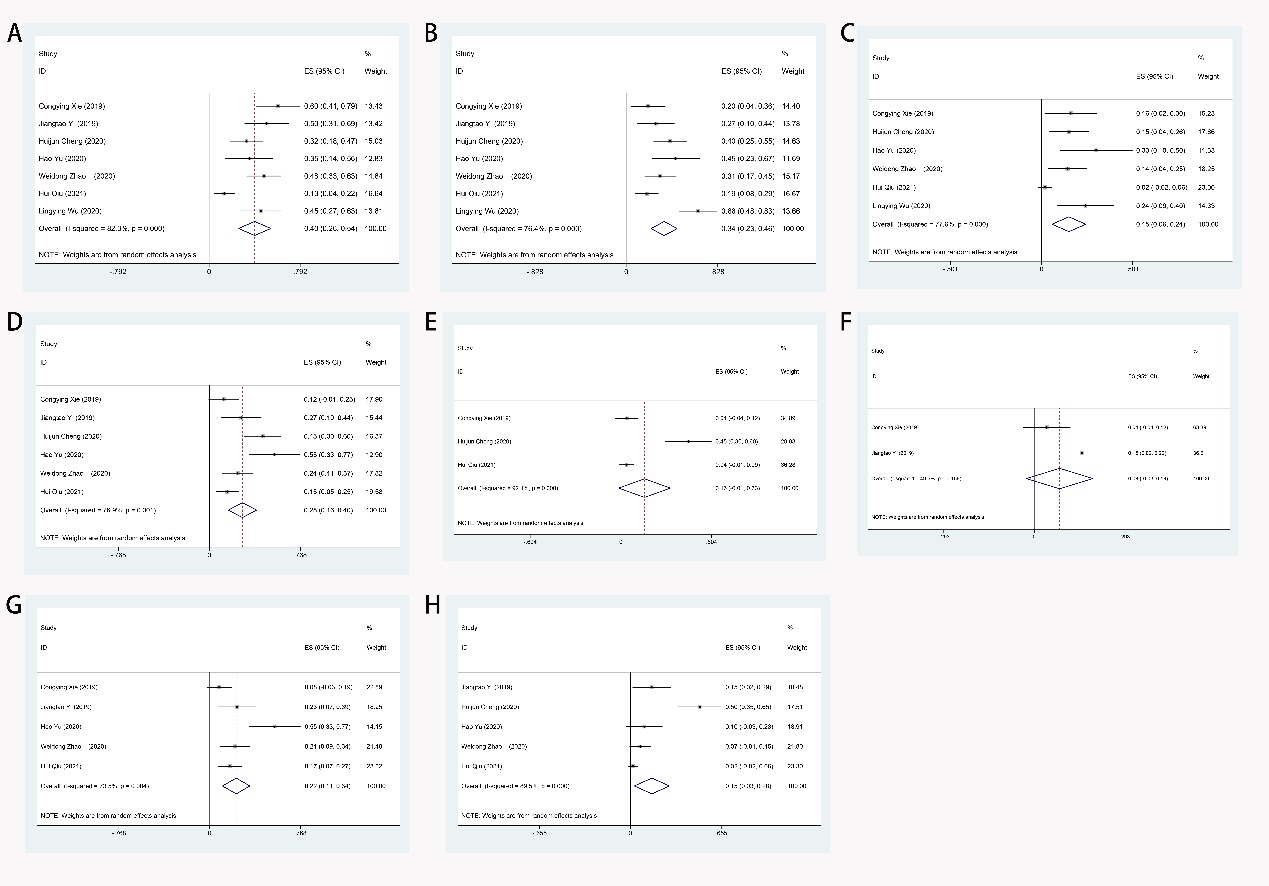


Figure. S1 The forest plot about the pooled results of Any Grade adverse events. (A) hand-foot syndrome (B) hypertension (C) proteinuria (D) fatigue (E) hemorrhage (F) thrombocytopenia (G) diarrhea and nausea (H) neutropenia.
